# A New Microwave Sensor Based on the Moore Fractal Structure to Detect Water Content in Crude Oil

**DOI:** 10.3390/s21217143

**Published:** 2021-10-28

**Authors:** Russul Khalid Abdulsattar, Taha A. Elwi, Zaid A. Abdul Hassain

**Affiliations:** 1 Electrical Engineering Department, Mustansiriyah University, Baghdad 1004, Iraq; eema1028@uomustansiriyah.edu.iq (R.K.A.); zaidasaad_79@uomustansiriyah.edu.iq (Z.A.A.H.); 2 Communication Engineering Department, Al-Ma’moon University College, Baghdad 1004, Iraq

**Keywords:** microwave sensor, Moore fractal structure, water content, crude oil

## Abstract

This paper presents a microwave sensor based on a two-ports network for liquid characterizations. The proposed sensor is constructed as a miniaturized microwave resonator based on Moore fractal geometry of the 4th iteration. The T-resonator is combined with the proposed structure to increase the sensor quality factor. The proposed sensor occupies an area of 50 × 50 × 1.6 mm^3^ printed on an FR4 substrate. Analytically, a theoretical study is conducted to explain the proposed sensor operation. The proposed sensor was fabricated and experimentally tested for validation. Later, two pans were printed on the sensor to hold the Sample Under Test (SUT) of crude oil. The frequency resonance of the proposed structure before loading SUT was found to be 0.8 GHz. After printing the pans, a 150 MHz frequency shift was accrued to the first resonance. The sensing part was accomplished by monitoring the S-parameters in terms of S_12_ regarding the water concentration change in the crude oil samples. Therefore, 10 different samples with different water percentages were introduced to the proposed sensor to be tested for detecting the water content. Finally, the measurements of the proposed process were found to agree very well with their relative simulated results.

## 1. Introduction

Moisture content detection in the crude oil derivatives is an important process of quality control evaluation because water is one of the most polluting materials in crude oils [1]. It is a requirement in the oil industry to minimize the water content as it is related to corrosion issues in downstream processing units as well as costs of transportation, safety issues, and economic disruption that might result from high water levels. For this reason, a fairly accurate measurement of water in crude oil is an essential requirement [2]. In [2], a procedure for detecting the moisture content in crude oil was proposed using microwave technologies. Later, the microwave technologies were extended by the researchers in [3,4] to enhance the method of water detection in crude oil. In [5], the water content in crude oil was determined using electrical conductivity measurement by using a voltage source to measure the response through current monitoring. Ultra-short-wave technology was applied to measure the water content in crude oil, as discussed in [6]. A spectral absorption method was proposed in [7] to measure water content in crude oil. A sensing method based on a capacitance to phase angle conversion method was proposed in [8] for water content measurements in crude oil in the real and short time during the production process.

Veselago introduced metamaterials for the first time in 1968 [8]. It became an interesting field for researchers because of their nontraditional features such as negative permittivity and/or permeability [9,10,11,12]. Since then, several microwave resonators based on metamaterials have been suggested by researchers for different applications at different operating frequencies. Recently, metamaterial resonators were widely used in evaluating the dielectric properties of substrates in terms of permittivity and loss angle tangent [13,14], which were applied in the characterization of liquid mixtures, such as the ratio of ethanol to water [15,16]. In [17,18], microwave sensors were proposed for oil quality testing. Microwave sensors were proposed as excellent candidates for many applications in the biomedical and industrial fields [19,20]. Usually, liquid characterization techniques-based microwave methods operate within the frequency bands from 0.5 GHz to 10 GHz. Metamaterial based on a microwave sensor attracted researchers attention due to their unique characteristic’s performance, such as their high sensitivity, small size, low cost, and ease of fabrication. The most common metamaterial resonance structure is shaped by split-ring resonators (SRRs), complementary SRRs (CSRRs) [21,22,23,24,25]. CSRRs and SRRs exhibit a well-established electrical field set up along the metamaterial structure that induces changes in the resonant frequency and Q-factor when the sensor is exposed to dielectric materials [24].

This study introduced a rectangular Moore fractal geometry based on a microwave resonator for crude oil characterization. The resonator consists of a 50 Ω transmission line that is loaded with a fractal Moore-shaped SRR. The proposed sensor is found to be very useful to detect the percentage of moisture contents in crude oils. The fractal geometry is designed to produce a resonant frequency at 0.8 GHz for the proposed microwave sensor circuitry. Nevertheless, the fractal geometry is invoked to improve the selectivity by generating a large area for the electric field fringing that increases the effective interaction area with the Sample Under Test (SUT). The sensor performance is tested experimentally with different water percentage introduction to the crude oil. According to the measurements, the water percentages with respect to the crude oil are recognized successfully with an overall error of less than 6%. The considered SUT are filled in the FR4 cartage pan that is mounted on the sensor surface to be directly exposed to the electric field fringing from the fractal geometry. A vector network analyzer (VNA) is connected to the sensor to measure the transmission spectra (S_21_) with different SUTs introduction. Finally, the theoretical results of the proposed sensor performance are validated experimentally to show excellent agreement with their relatives.

## 2. Geometric Details of the Sensor

**Figure 1:**
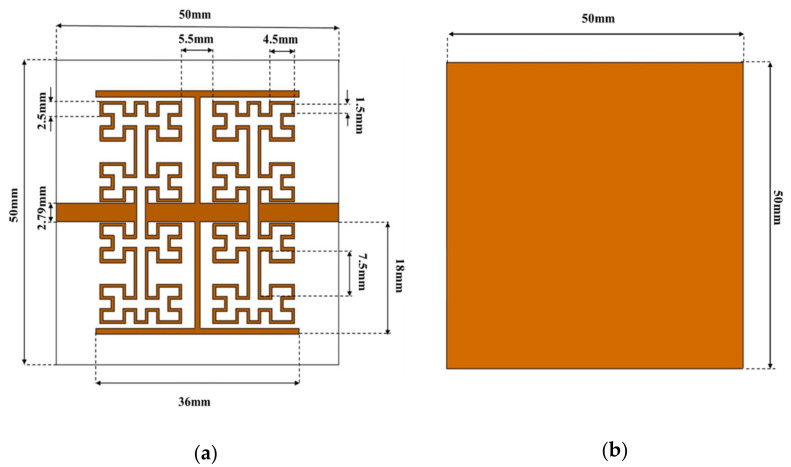
The proposed sensor geometrical details: (**a**) front view and (**b**) back view.

In this section, the authors discussed their proposed sensor structure, see Figure 1a, based on Moore fractal geometry. The sensor is designed based on three main parts mounted on an Epoxy Glass FR4 substrate. The first part is constructed as a transmission line with two transverse slots as air gaps to realize a capacitive coupling to ensure the field fringing mechanism [26]. Next, four Moore-shaped inclusions as high capacitive surfaces [27]. were introduced to the design to ensure the field leakage over a wide region from the SUT. For this, the induced electric field intensity between the transmission line and SUT would be magnified significantly [28] to ensure deep penetration in the SUT. The last part that is represented by the T-shaped resonator, was introduced to maintain the frequency resonance of the proposed sensor toward the lower frequency band of interest [29]. The resonator layer from the front view of the substrate was made from copper, and it has a thickness equal to 0.035 mm. The authors designed their sensor to operate around 0.8 GHz to obtain excellent penetration through the skin depth criterion [30]. On the other hand, a significant increase in the effective permittivity (ε_r_) would be achieved by adding the T-shaped resonator to the Moore inclusions. Thus, the induced electric field intensity [31] would be enhanced for the SUT dielectric properties characterization. The proposed sensor is backed with a full ground plane based on a copper layer, as seen in Figure 1b.

It is good to mention that the proposed Moore geometry dimensions are calculated to achieve a frequency resonance around 0.8 GHz. Moore-1 is the term for the fundamental element. Moore-n is a new fractal structure combined to form n copies of Moore-1, and by inserting one minimal fractal segment in the joint between two nearby components, the novel fractal structure of Moore-n is developed. The required number of segments that are applied in the proposed Moore-n (*N_n_*) structure is determined by [31].

(1) $$N_{n}=nN_{1},$$

and total length $L^{n}$ extends with each operation and is calculated as:

(2) $$L^{n}=\frac{8n}{2n+3}L_{0}^{n},$$

where *N*_1_ is the number of fractal segments of Moore-1 and $L_{0}^{n}$ is the perimeter of a conventional rectangle occupying the same area with its corresponding Moore-n. Suppose the curve filled in a square section *S* as external side by increasing the number of generations, the area between lines diminishes, therefore, and the length of total perimeter increases as:

(3) $$L\left(n\right)=\left(2^{n}+1\right)S,$$

From (3), the perimeter is found to be exponentially increased with the *n* incremental.

## 3. Moore Cell Characterizations

The proposed Moore structure is constructed from a fractal geometry of the 4th iteration, as seen in Figure 1a. The outer area of the unit cell is considered 16.5 × 14.5 mm^2^. The proposed fractal is constructed from a polygonal conductive trace of 0.5 mm width. Such width is considered to avoid the cross lines intersections and radiation leakage from the trace width [27]. Therefore, to characterize the proposed fractal when mounted on the FR4 substrate in terms of dispersion diagram and S-parameters, 3D full-wave analysis was conducted based on the CST MWS software package. In such a study, the authors conducted their analysis parametrically.

**Figure 2:**
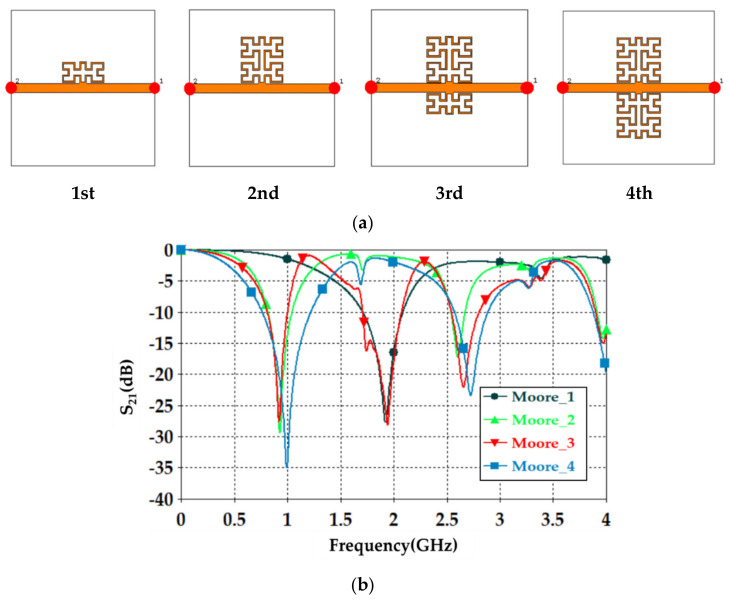
Parametric study based on changing Moore iteration: (**a**) Moore iteration around the transmission line geometrical models. (**b**) S_21_ spectra variation with respect to the Moore iteration change.

Now, the effects of changing the Moore iteration with respect to the proposed unit cell performance were studied. Therefore, the slots on the transmission line were not considered for an instant. The configuration and distribution of the unit cell around the proposed transmission line center are shown in Figure 1a. S_21_ spectra were monitored with respect to fractal iteration increase, as displayed in Figure 2a. As seen from S_21_ spectra in Figure 2b, the first iteration for the proposed geometry showed a single frequency band around 2 GHz and S_21_ about −27 dB. However, increasing the Moore order to the 2nd iteration created two frequency bands around 1 GHz, S_21_ about −29 dB and 2.5 GHz, S_21_ about −17 dB. At the 3rd iteration, three frequency modes appeared within the frequency band of interest due to the coupling effects between the unbalanced distributions around the unit cell axis [32], which was not desired because energy dissipation could happen in such a mode that leads to measurement distortion effects. The 4th iteration was found to show two modes at 0.9 GHz, S_21_ about −35 dB and 2.75 GHz, S_21_ about −23 dB as seen in Figure 2b. Therefore, the authors decided to consider the 4th iteration for the proposed sensor design because the lowest S_21_ magnitude was found in the 4th iteration. This is usual in microwave resonators based on two-port networks. When the system is a lossless network in such devices, |S_11_|^2^+|S_12_|^2^ = 1. Otherwise, the system would be a lossy system.

**Figure 3:**
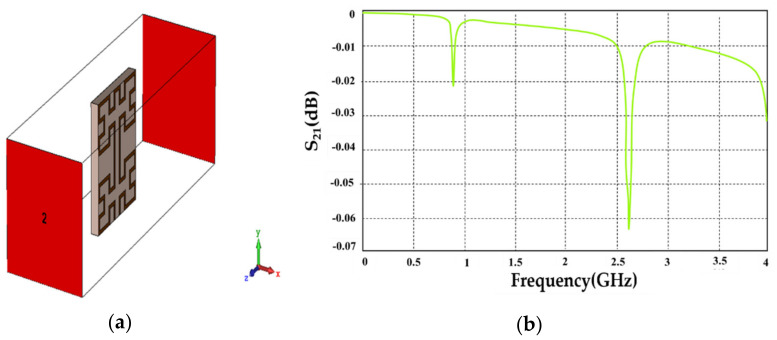
Unit cell performance (**a**) unit cell location inside the waveguide and (**b**) S_21_ spectra.

Next, S_21_ spectra were evaluated for an individual unit cell based on the 2nd iteration using a square cross-sectional area waveguide to mimic the plane wave propagation based on the effective medium theory [8]. The unit cell was located at the center of that waveguide; see Figure 3a, to evaluate the S_21_ spectra. The evaluated S-parameters are presented in Figure 3b with respect to fractal iteration variation. It was found that the proposed unit cell showed two fundamental modes around 1 GHz and 2.5 GHz; to agree with the previous results of two unit cells and four-unit cells study. Such results encouraged us to conduct the proposed sensor design based on a balanced geometry in symmetrical configuration to maintain the resonance around the fundamental modes.

## 4. Design Methodology and Systematic Study

**Figure 4:**
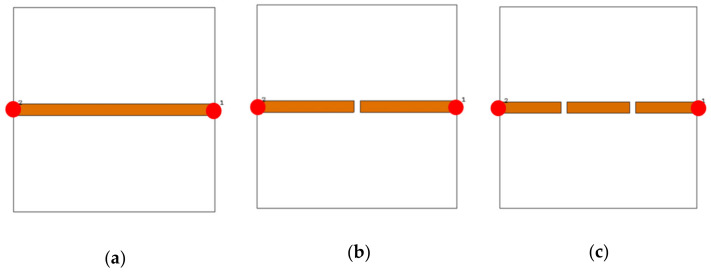
Transmission line structure: (**a**) without slot, (**b**) single slot, and (**c**) dual slots.

**Figure 5:**
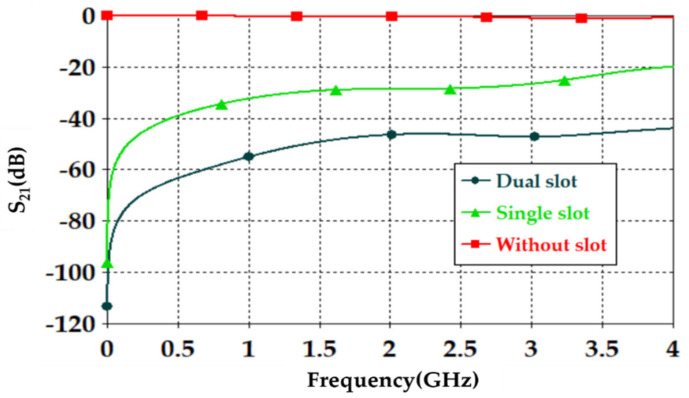
S_21_ spectra for the proposed transmission line.

**Figure 6:**
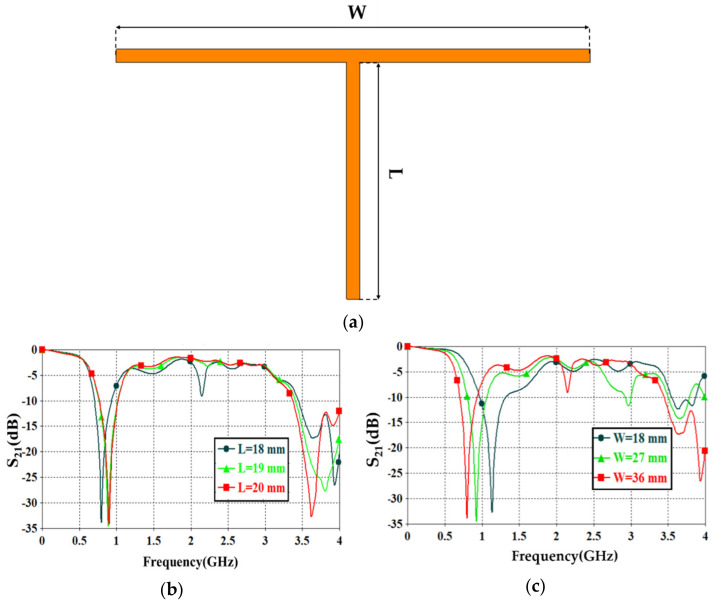
(**a**) The proposed T-resonator (**b**) S_21_ spectra of the proposed unit cell with respect to the T-resonator dimensions change length variation and (**c**) S_21_ spectra of the proposed unit cell with respect to the T-resonator dimensions change width variation.

**Figure 7:**
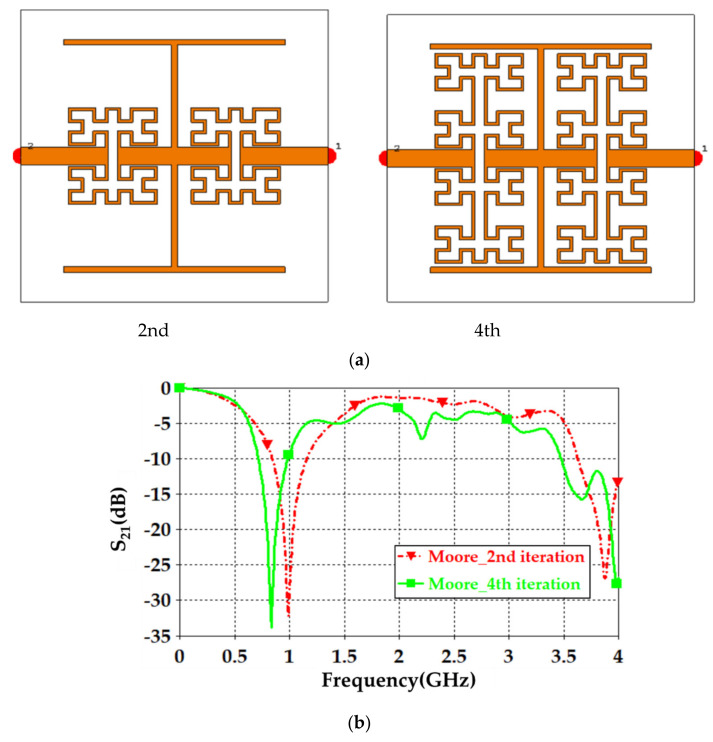
(**a**) The proposed sensor Moore unit cells based on 2nd and 4th iterations (**b**) S_21_ spectra for 2nd and 4th iterations.

**Figure 8:**
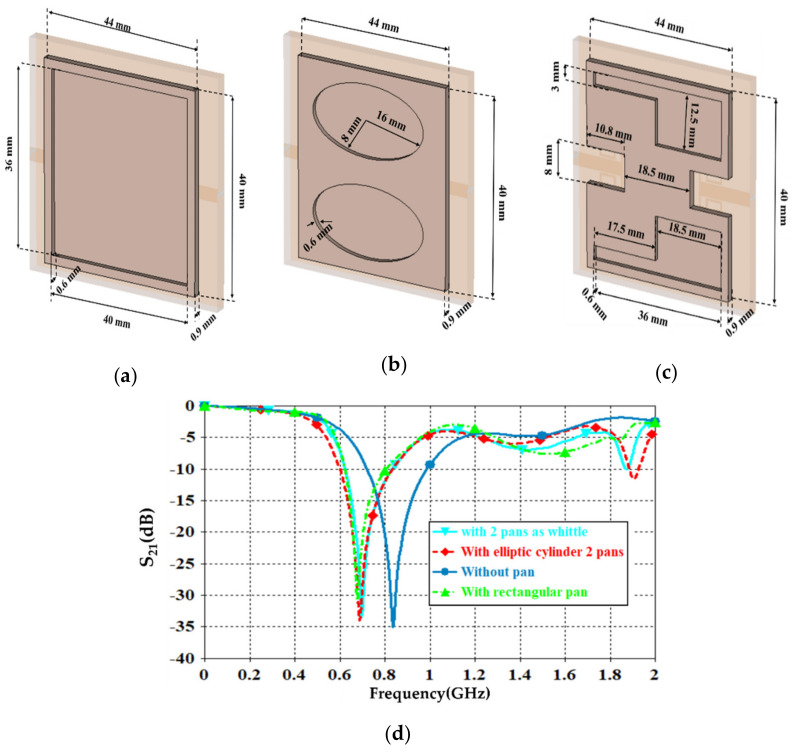
Pans models: (**a**) rectangular pan (**b**) elliptic cylinder pan (**c**) whittle pan, and (**d**) S_12_ spectra.

**Figure 9:**
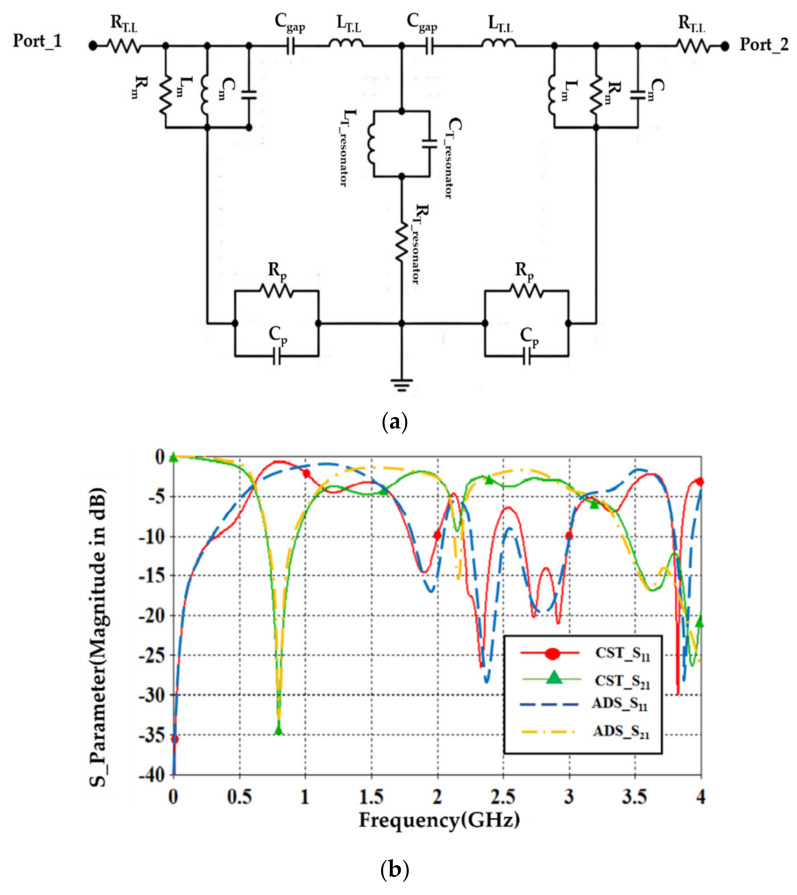
Circuit analytical model: (**a**) circuit model and (**b**) S-parameters.

The authors proposed their design development in this section to visualize the conducted approach to reach the optimal performance for the operation. Therefore, the design process was broken down into the following steps:

A. Transmission line design
  First of all, the authors designed a 50 Ω transmission line, see Figure 4, to ensure power motion fluently from port 1 to port 2. Next, a single slot was introduced to the transmission line to leak the electromagnetic fringing in a specific area [13]. Such fringing increased the field penetration to the SUT, as will be seen later. Therefore, 2 slots were included instead of 1 slot around the transmission line center to increase the field fringing.
  Now, S_21_ spectra were evaluated using CST MWS for the frequency bands from 0.1 GHz up to 4 GHz. As seen in Figure 5, it was found that introducing a single-slot reduced S_21_ significantly over the entire frequency band of interest. However, increasing the slot number to dual slots reduced S_21_ rapidly below −40 dB. Such a reduction in the magnitude of S_21_ value would be invested for the detection process. Nevertheless, such slots created capacitive coupling to store the electromagnetic energy at high frequencies [33]. It is good to mention that, for microwave resonator design, the frequency resonances can be observed from the S_12_ spectra, as explained in [1].
B. T-resonator effects
  Now, the proposed T-resonator, as seen in Figure 6a, performance in terms of S_21_ spectra was evaluated parametrically by changing the trace line length and the transmission line length. Therefore, trace length (L) was changed from 18 mm to 20 mm with a step of 1 mm. Such length was limited to these values to avoid any intersection between the conductor parts of the proposed sensor. In general, it was found that the proposed T-resonator showed a frequency resonance around 0.8 GHz that was very close to the resonance of the proposed fractal unit cell. However, the second mode was found far away from the second mode of the proposed Moore, as seen in the previous section. Such conclusions motivated us to consider the first mode for the sensing process only. Nevertheless, it was found that when the T-resonator length was 18 mm, the frequency resonance was shifted to 0.8 GHz, as seen in Figure 6b. However, the frequency resonance at the first mode, around 0.9 GHz, was found to be unaffected by increasing the length after 19 mm. Therefore, the T-resonator length was considered 18 mm for the proposed cell geometry. Then, the transmission line length (W) was changed from 18 mm to 36 mm with a step of 9 mm. We found from Figure 6c that the proposed structure showed a frequency resonance around 0.8 GHz, which was the frequency band of interest at 36 mm. However, the other two lengths, at 18 mm and 27 mm, showed frequency resonances out of the frequency band of inters. Therefore, in the proposed design, the length of the transmission line was fixed to 36 mm.
C. Moore unit cell introduction
  The proposed Moore unit cells based on the 2nd and 4th iterations, see Figure 7a, were only introduced to the sensor design by placing four of them connected directly to the transmission line at the slots locations. The proposed study was considered to ensure the effects of changing the iteration order in the final design stage through monitoring the S_21_ spectra for the two cases, as seen in Figure 7b.
  It would be very important to emphasize that the authors’ consideration of the 4th iteration in their final design for two main reasons: first, increasing the field intensity by increasing the fractal iteration that was very desirable for the detection process [32]. In addition, the 4th iteration generated a frequency resonance around 1 GHz that would be shifted to the lower frequencies after introducing the pans and the SUT. This makes the proposed sensor operation in the range of 0.8 GHz that was a design specification for this work. As well as maintaining the sensor operation with a low-frequency band, which is very useful for field penetration inside the SUT [32].
D. Shapes of Pans
  This section discusses the effects of introducing different shapes of pans to select the suitable geometry of the proposed sensor. The pans should cover the area of the Moore structure to ensure the field penetration to the SUT. The first suggested pan shape, see Figure 8a, assumed a rectangular geometry with a size of 40 × 44 × 0.6 mm^3^. The second shape was considered as an elliptic cylinder that was scratched from a rectangular area with a height of 0.6 mm, as seen in Figure 8b. Another shape of the pan was suggested by using a rectangular plate having a compact size equal to 40 × 44 × 0.6 mm^3^ with a whittle shape, as presented in Figure 8c. This geometry was assumed based on the observed electric field intensity distributions that will be seen later. It is good to mention that all suggested geometries have the same height, 0.6 mm, to avoid any discrepancy during the design. In addition, this value was assumed based on the suggested height from [11,34] that agrees well with our parametric study, as will be shown later.
  From the simulation results using CST MWS, it was found that the proposed sensor based on rectangular pans provided S_21_ of −31 dB with a resonant frequency shift of 156 MHz, as seen in Figure 8d. After introducing the other pans to the proposed sensor, S_21_ was found to be −33 dB with a resonant frequency reduction of around 148 MHz from the 0.8 GHz, as depicted in Figure 8d. The simulated results, see Figure 8d, show that the proposed sensor based on the whittle shape provides S_21_ equal to −34 dB with a resonant frequency shift of 140 MHz with respect to 0.8 GHz. Therefore, we considered the whittle shape pans in our next study; because it provides us the minimum frequency shift.
E. Equivalent circuit model
  The proposed sensor geometry based on the T-resonator and fractal Moore geometry of the 4th iteration was analyzed analytically using an equivalent circuit model. We derived the equivalent circuit model of the proposed structure based on the lumped elements Richard model [35]. As seen in Figure 9a, the proposed circuit model was considered by connecting a 50 Ω input impedance RF source in series with an (R-L-C) parallel branch. The R-L-C branch was denoted for Moore geometry to be named as L_m_, R_m_, and C_m_. The main transmission line was characterized by an inductive part L_T_ and a capacitive air gaps C_gap_ that was shown previously in Figure 1. The T-resonator as a load was connected to the center of the transmission line and defined as L_T-resonator_ in parallel with a capacitor of C_T-resonator_. This branch was connected serially with a resistor of R_T-resonator_. Each of R_p_ and C_p_ was connected in parallel with the equivalent circuit model to denote the pans effects. The S-parameters were evaluated for the proposed circuit model and compared to those obtained from CST MWS. A good agreement was achieved according to the listed lumped elements, which were simulated in Adjulent Devices Simulator (ADS), as seen in Table 1.

## 5. Numerical Analysis

**Figure 10:**
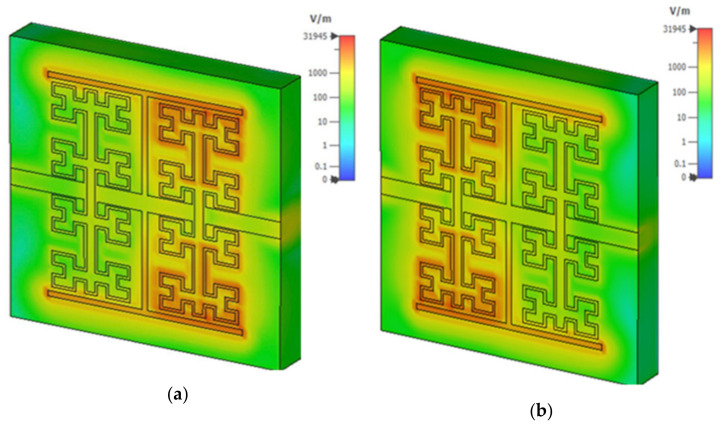
Electric field simulation at the resonance frequency: (**a**) from the first port and (**b**) from the second port.

The proposed sensor was designed to characterize liquids when placed on the fractal part of the sensor in the pans that were made from the same substrate material FR4. When the resonances occur, the total electric field is mainly concentrated in the fractal geometry regarding the first port, as seen in Figure 10a, which is coupled capacitively to each side of the transmission line. However, the field was effectively degraded to the second port, as seen in Figure 10b, due to the effects of band rejection of the T-resonator. Such behavior was considered for the proposed work to avoid the measurements discrepancy due to field retardation from the second port [29,35].

**Figure 11:**
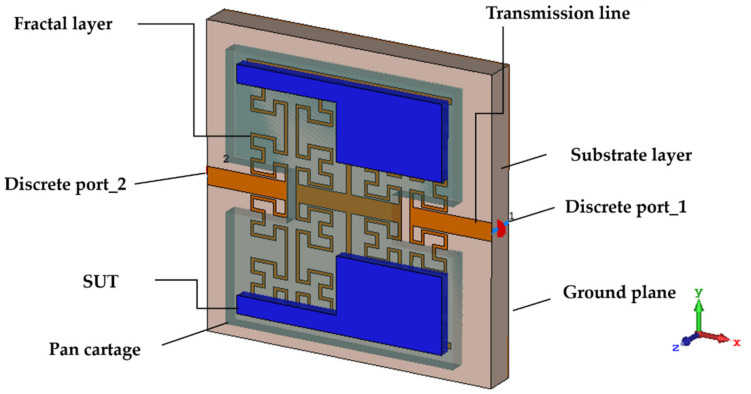
Sensor with the sample under test placed on the fractal structure.

Now, from the obtained field distribution, we suggested the shape of the pan cartage that contains the liquid, as seen in Figure 11. This is considered according to the confined electric field from Figure 10 to detect any change in the effective permittivity of the SUT. As will be noticed later, the responses of the proposed sensor, the resonant frequency, and the quality factor, were found to be significantly changed with the variation of the effective permittivity. This could happen through the electric field perturbation to ensure the resonance frequency shift according to the SUT dielectric properties [36].

**Figure 12:**
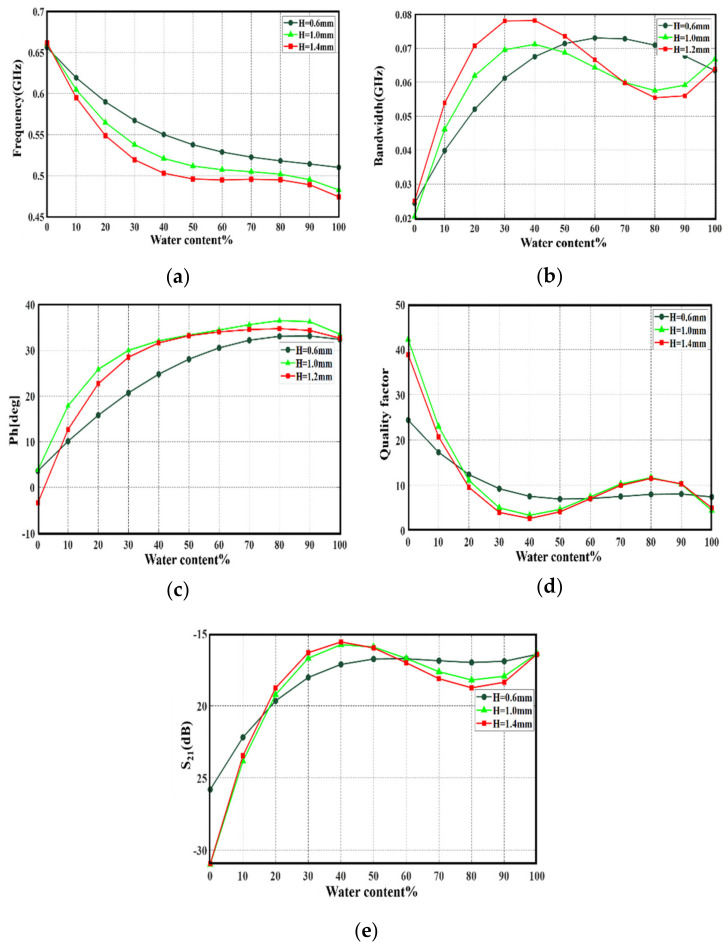
S_21_ variations with different pan heights in terms of: (**a**) resonance frequency (**b**) bandwidth (**c**) phase (**d**) quality factor, and (**e**) S_21_ magnitude.

Next, through full-wave electromagnetic simulation in CST MWS environments, we investigated the resonant frequency changes after loading the sensor with crude oil samples. Therefore, S_21_ spectra were monitored to quantify the resonant frequency variation with respect to the unloaded sensor response. This study mixed a certain volume of the crude oil with different percentages of water introduction to be changed from 0% to 100%. However, due to the phenomena of oil floating on the top of the water, because the density of water is higher than the density of oil [37], numerically, the water layer is mounted underneath the oil layer. In the same context, the water and oil surface areas have the same surface area of the pan. Therefore, any changes in the amount of water to oil percentage are relative to the liquid height only. Thus, the resulting variations in the S_21_ spectra were measured with respect to the water height change indeed. On the other hand, since the proposed sensor is considered for liquids characterizations, the pan height must not exceed a few tenths of the guided wavelength to avoid field retardation from the boundaries [11]. Therefore, a parametric study was considered for three different highest of the substrates by changing it to 0.6 mm, 1 mm, and 1.4 mm. From the monitored S_21_ spectra, the variation in the frequency resonance, bandwidth (B.W), phase, and quality factor (Q) with respect to the water percentage change and pan height are recorded in Figure 12. It was found that with increasing the pan height, most recorded variations conflicted with the nonlinear slope. Thus, the authors considered 0.6 mm height as the best choice to avoid readings discrepancy due to the nonlinear variations. In which, 0.6 mm height performed the most variation results without conflict points, curve maxima or minima, at the resonant frequency and phase change. For more details about varying the water percentage with respect to the oil, Table 2 shows more specific details about the other cases.

**Figure 13:**
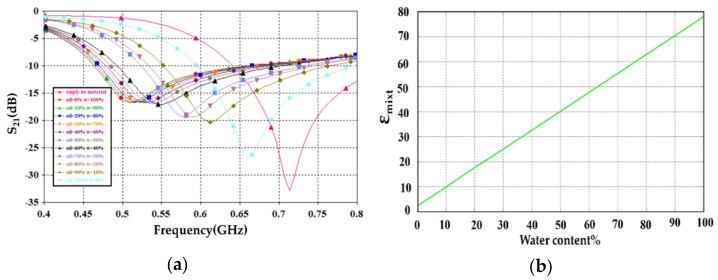
Variation in the water percentage with respect to the oil: (**a**) S_21_ spectra and (**b**) effective permittivity variation.

Next, the variations in S_21_ spectra in specific at 0.6 mm pan height were evaluated numerically and presented in Figure 13a. It was observed that the resonant frequency mostly shifted to lower frequency bands by increasing the water percentages. This was because the effective mixture’s effective permittivity value increased rapidly with increasing the water percentage, according to Figure 12b. In Figure 12b, the change in the dielectric constant of the water-oil mixture was calculated from the following relationship [34]:

(4) $$\varepsilon_{mixt}^{a}=\sum_{i}f_{i}\;\varepsilon_{i}^{a},$$

where the permittivity of the mixture $\varepsilon_{mixt}$ as a function of the permittivities of the constituents $\;\varepsilon_{i}^{a}$ through Equation (4), where f_*i*_ is the volume fraction of the ith constituent.

The calculation of the frequency resonance change, bandwidth, phase, and Q-Factor are basically the functions of water percentage variation in crude oil. This is associated with changing the concentration of the water-oil mixture percentage. Based on the curve fitting model, a polynomial relationship is given by (5) to describe resonance frequency change with respect to water content in the SUT:

(5) $$f\left(x\right)=p_{3}x^{3}+p_{2}x^{2}+p_{1}x+p_{0},$$

where x water percentage in crude oil.

The factors $p_{0}$,$\;p_{1}$,$\;p_{2}$, and $p_{3}$ given in (5) are unknown coefficients that will define a complete relation of dependence resonance frequency with water content of samples, once they are calculated accurately. From Figure 12a, we can solve the Equation (5) by curve fitting in MATLAB and find unknown variables as following:

$p_{0}=$ 0.5376
$p_{1}=$ −0.03488
$p_{2}=$ 0.01997
$p_{3}=$ −0.005978

The model describing changes in the phase with respect to water content variation in the SUT is defined by the following polynomial equations:

(6) $$Ph\left(x\right)=p_{3}x^{3}+p_{2}x^{2}+p_{1}x+p_{0},$$

The factors $p_{0}$,$\;p_{1}$,$\;p_{2}$, and $p_{3}$ given in (6) are unknown coefficients that define a complete relation of dependence of phase with water content variation in the SUT. From Figure 12b, it is found:

$p_{0}=$ 28.03
$p_{1}=$ 5.877
$p_{2}=$ −4.424
$p_{3}=$ 2.266

For, the bandwidth variation with respect to water content change in the SUT, the polynomial is found to be as:

(7) $$B.W=p_{2}x^{2}+p_{1}x+p_{0},$$

where

$p_{0}=$ 0.07138
$p_{1}=$ 0.01182
$p_{2}=$ −0.0121

The moisture content in crude oil was obtained by compensation measured values in practical as shown in Table 3 and values of coefficient that measured by curve fitting in MATLAB and compensation in equations above from (5 to 7).

## 6. Fabrication and Experimental Validation

**Figure 14:**
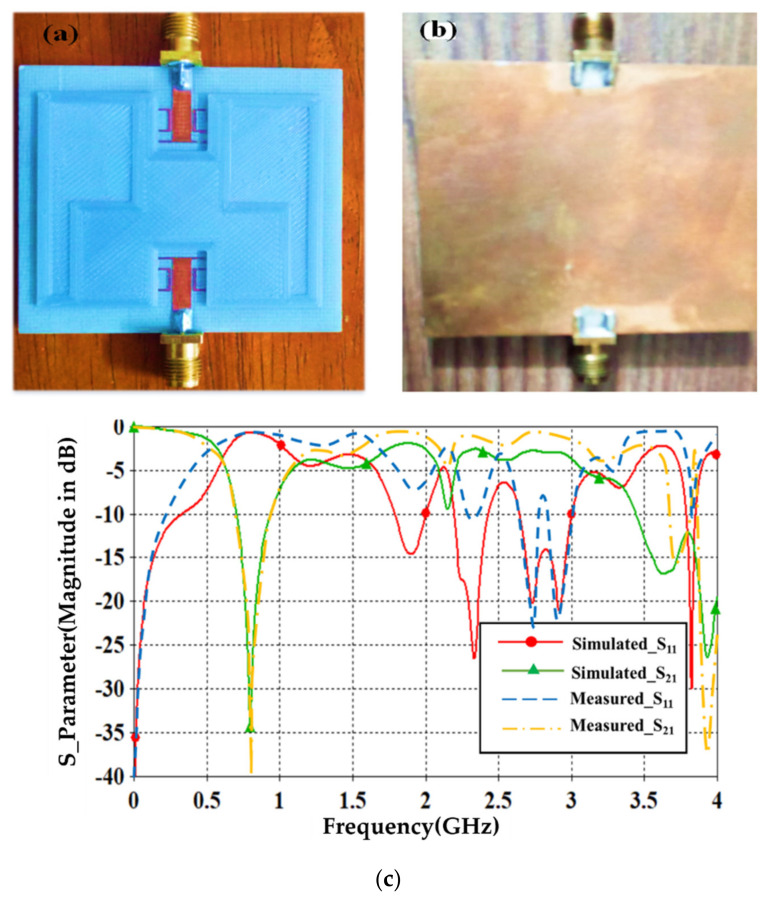
Experimental validation: (**a**) fabricated sensor front view, (**b**) fabricated sensor back view, and (**c**) S_Parameter spectra.

The proposed sensor was fabricated and measured experimentally in terms of S_11_ and S_21_ spectra. The fabricated sensor is presented in Figure 14a,b. The proposed sensor was measured using a Professional Network Analyzer (Agilent PNA 8720) after applying a through-transmission calibration process. Nevertheless, a two ports calibration was applied to port_1 and port_2 using open, short, and load processes. The measurements were conducted to S_11_ and S_21_ spectra, as depicted in Figure 14c. The calibration was applied before measurements to eliminate any possible errors based on the mechanical calibration kit: open, short, load, and thru. We found that the proposed sensor showed a well-defined resonance at 0.78 GHz with S_21_ = −40 dB. For the measurements, 10,000 points were utilized with –10 dBm input power with 0.1 dB of S_21_. An excellent agreement was found between the numerical results and measurements in terms of S_11_ and S_21_ spectra within the frequencies from 0.1 GHz up to 4 GHz. However, the insignificant discrepancy between measurements and simulated results were found to be less than 6%. Such discrepancy could be attributed to the effects of soldiering and fabrication errors, as well as the decoupling between the RF SMA ports and the edge of the copper trace [38].

After the sensor fabrication, the simulation results were compared to the measured results without SUTs. Now, the sensor was tested by taking different samples of the crude oil and water mixture and measured experimentally in terms of S_21_ spectra. The measured results were found to agree well with the simulated results for all cases.

From Table 3, the measurements in terms of frequency resonance, phase variation, quality factor, and the least mean square error of S_21_ between the simulation and measured results are listed. We found that the proposed sensor showed the shift in resonate frequency when the water percentage from (3–76%) of about 0.144 GHz. After that, the proposed sensor had fewer effects on increasing the water percentage because the relative permittivity of water was about 80 and 2.44 for the crude oil. Therefore, the relative permittivity of the water-continuous combination was dominated by water content [39].

Finally, this work contributes to designing a new design microwave sensor for moisture detection in crude oil, which works at a low-frequency band. The proposed sensor is based on Moore’s 4th order fractal geometry because the fractal characteristics may be used to design components with specific electromagnetic properties. For the same resonant frequency needs, fractal resonators were proven to be considerably smaller than planar resonators, the size of the resonators decreased, and the quality factor was greater than 50. The quality factor increased as the number of fractal iterations increased. Table 4 shows the performance comparison between the proposed sensor of this work and other microwave sensors for other researchers:

## 7. Conclusions

The proposed sensor successfully detects various percentages of pollution of water in crude oil. The proposed sensor is realized on FR-4, while the pans on the sensor surface are made of the same material as the substrate. The proposed sensor operates at about 0.8 GHz. This system consists of placing SUT in the pans. The frequency resonance of the proposed sensor is found to be significantly affected after introducing different SUT because the effective permittivity variation of the SUT with water contents change. Therefore, to validate the proposed sensor performance, a measurement study was applied to observe a distinguishable resonance variation with respect to the water concentration change from 0% to 100%. The proposed sensor size was miniaturized to 50 × 50 mm^2^ with low-cost, non-contact, and reusable properties. This makes the proposed sensor a very suitable candidate for monitoring low concentrations, about 10%, of water using extremely small liquid volume, as validated experimentally by conducting 10 samples. In future work, we are planning to use the proposed sensor on a flexible substrate for wearable biomedical devices due to their growing demands in the industry. Therefore, we are planning to test this sensor with different biological fluids.

## Figures and Tables

**Table 1:** Lumped elements based on the circuit model of the proposed sensor.

R_T_._L_	L_T_._L_	L_m_	C_m_	R_m_	C_gap_	L_T-resonator_	C_T-resonator_	R_T-resonator_	C_P_	R_P_
50 Ω	9 nH	0.1 nH	2 pF	10 Ω	1.3 pF	8nH	6.1 pF	101 Ω	0.5 pF	15

**Table 2:** More specific details about the other relative cases.

Water%	Oil%	fr(GHz)	Δfr(GHz)	S_21_(dB)	B.W(GHz)	Ph[deg]	Q.F
0	100	0.66	0	−26.836	0.025926	−0.510076	25.45707
10	90	0.612	0.04	−20.346	0.038896	14.97738	15.73426
20	80	0.588	0.072	−19.405	16.615898	16.615898	11.13172
30	70	0.576	0.084	−19.278	18.615898	18.615898	11.29633
40	60	0.546	0.114	−17.109	24.823846	24.823846	7.19737
50	50	0.54	0.12	−16.882	33.194263	33.194263	7.20393
60	40	0.528	0.132	−16.767	28.492114	28.492114	7.19159
70	30	0.522	0.138	−16.738	29.431511	29.431511	7.738477
80	20	0.516	0.144	−16.690	28.793417	28.793417	7.47847
90	10	0.5159	0.1444	−16.713	36.325119	36.325119	7.78204
100	0	0.51	0.15	−16.722	33.350711	33.350711	7.79387

**Table 3:** Practical measurements of different mixtures percentage using the proposed sensor.

Water%	Oil%	fr(GHz)	Δfr(GHz)	S_21_(dB)	B.W(GHz)	Ph[deg]	Q.F	Error%
3	97	0.671	0.0001	−28.886	0.025	19.915	26.84	0.063
11	89	0.609	0.051	−19.617	0.051	9.076	11.94	0.055
14	86	0.608	0.059	−14.554	0.055	17.161	11.054	0.156
21	79	0.58	0.069	−14.451	0.052	14.981	11.153	0.144
23	77	0.582	0.075	−13.393	0.054	14.706	10.778	0.1787
28	72	0.575	0.081	−19.278	0.058	28.292	9.914	3.064 × 10^−3^
34	66	0.574	0.084	−21.803	0.049	23.428	11.714	0.059
40	60	0.546	0.112	−17.392	0.076	23.996	7.184	0.057
46	54	0.541	0.114	−14.992	0.078	23.956	6.936	0.069
52	48	0.54	0.124	−17.052	0.073	34.263	7.397	5.01 × 10^−3^
55	45	0.527	0.133	−15.986	0.079	27.969	6.671	0.025
61	39	0.527	0.131	−17.716	0.077	32.335	6.844	0.027
66	34	0.523	0.141	−15.928	0.073	27.916	7.164	0.027
71	29	0.521	0.14	−16.767	0.07	29.049	7.442	1.875 × 10^−3^
72	28	0.516	0.143	−13.493	0.072	29.385	7.167	0.108
76	24	0.516	0.144	−17.071	0.069	35.281	7.478	8.21 × 10^−3^
80	20	0.516	0.144	−15.907	0.071	36.091	7.268	0.028
85	15	0.516	0.144	−15.993	0.069	31.907	7.478	0.024
91	9	0.516	0.144	−15.532	0.067	35.319	7.701	0.035
95	5	0.513	0.148	−12.314	0.068	34.926	7.544	0.0172
100	0	0.511	0.149	−16.977	0.062	35.979	8.225	6.07 × 10^−3^

**Table 4:** Comparison between microwave sensors.

Ref.	Type of Resonators	f_r_ (GHz)	Area of Substrate (mm^2^)
[1]	Oval Wing Resonator	8–10	35 × 35
[15]	Complementary Circular Spiral Resonator	2.4	20 × 28
[16]	Complementary Split Ring Resonators	2.4	30 × 25
[23]	Multiple Complementary Split-Ring Resonator (MCSRR)	2.45	35 × 25
[24]	Split ring resonator	1.9	28 × 28
[40]	Cesaro Fractal Electromagnetic Bandgap Structure (EBG)	2.45	82 × 82
[41]	Fractal Peano Curve	4.494	75 × 25
This work	Moore Fractal Geometry	0. 8	50 × 50

## Data Availability

Not applicable.
